# Benzoic acid–2,2′-biimidazole (2/1)

**DOI:** 10.1107/S1600536810045368

**Published:** 2010-11-10

**Authors:** Xiaoli Gao, Miaoli Zhu

**Affiliations:** a Department of Chemistry, Taiyuan Normal College, Taiyuan, Shanxi 030031, People’s Republic of China; b Institute of Molecular Science, Key Laboratory of Chemical Biology and Molecular Engineering of the Education Ministry, Shanxi University, Taiyuan, Shanxi 030006, People’s Republic of China

## Abstract

In the title compound, C_6_H_6_N_4_·2C_7_H_6_O_2_, the asymmetric unit contains a half-mol­ecule of biimidazole and one benzoic acid mol­ecule. The unit cell contains two biimidazole mol­ecules and four benzoic acid mol­ecules, giving the reported 2:1 ratio of benzoic acid to biimidazole. The biimidazole mol­ecule is located on an inversion center (passing through the central C—C bond). Strong N—H⋯O and O—H⋯N hydrogen bonds link the benzoic acid mol­ecules with the neutral biimidazole mol­ecules, which lie in planar sheets. In the crystal packing, the parallel sheets are related by a twofold rotation axis and an inversion centre, respectively, forming an inter­woven three-dimensional network *via* weak C=O⋯π inter­molecular inter­actions between neighboring mol­ecules.

## Related literature

For background to the use of 2,2′-biimidazoles in crystal engineering, see: Matthews *et al.* (1990 ▶); Tadokoro & Nakasuji (2000 ▶). For similar structures, see: Gao *et al.* (2009 ▶); Li & Yang (2006 ▶); Mori & Miyoshi (2004 ▶).
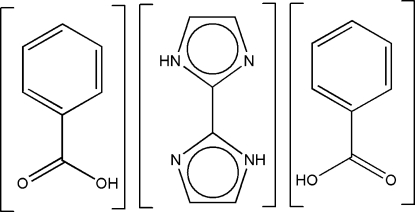


## Experimental

### 

#### Crystal data

C_6_H_6_N_4_·2C_7_H_6_O_2_
*M* *_r_* = 378.38
Monoclinic, 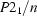

*a* = 11.232 (5) Å
*b* = 5.082 (2) Å
*c* = 16.342 (7) Å
β = 99.832 (6)°
*V* = 919.2 (7) Å^3^
*Z* = 2
Mo *K*α radiation
μ = 0.10 mm^−1^
*T* = 298 K
0.40 × 0.20 × 0.10 mm

#### Data collection

Bruker SMART 1K CCD area-detector diffractometer
Absorption correction: multi-scan (*SADABS*; Sheldrick, 2000 ▶) *T*_min_ = 0.962, *T*_max_ = 0.990
3367 measured reflections
1550 independent reflections
1243 reflections with *I* > 2σ(*I*)
*R*_int_ = 0.047

#### Refinement

*R*[*F* ^2^ > 2σ(*F* ^2^)] = 0.098
*wR*(*F* ^2^) = 0.188
*S* = 1.25
1550 reflections
131 parameters
H atoms treated by a mixture of independent and constrained refinement
Δρ_max_ = 0.20 e Å^−3^
Δρ_min_ = −0.19 e Å^−3^

### 

Data collection: *SMART* (Bruker, 2000 ▶); cell refinement: *SAINT* (Bruker, 2000 ▶); data reduction: *SAINT*; program(s) used to solve structure: *SHELXS97* (Sheldrick, 2008 ▶); program(s) used to refine structure: *SHELXL97* (Sheldrick, 2008 ▶); molecular graphics: *ORTEP-3* (Farrugia, 1997 ▶) and *SHELXTL/PC* (Sheldrick, 2008 ▶); software used to prepare material for publication: *SHELXTL/PC*.

## Supplementary Material

Crystal structure: contains datablocks I, global. DOI: 10.1107/S1600536810045368/fl2323sup1.cif

Structure factors: contains datablocks I. DOI: 10.1107/S1600536810045368/fl2323Isup2.hkl

Additional supplementary materials:  crystallographic information; 3D view; checkCIF report

## Figures and Tables

**Table 1:** Hydrogen-bond geometry (Å, °) *Cg*1 is the centroid of the N1/C1/N2/C3/C2 ring.

*D*—H⋯*A*	*D*—H	H⋯*A*	*D*⋯*A*	*D*—H⋯*A*
O1—H1*A*⋯N2^i^	0.86	1.77	2.613 (5)	170
N1—H1⋯O2^ii^	0.88 (5)	1.89 (5)	2.767 (5)	173 (5)
C4—O2⋯*Cg*1	1.22 (1)	3.67 (1)	4.388 (2)	118 (1)

Symmetry codes: (i) 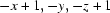; (ii) 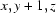.

**Fig. 1.**
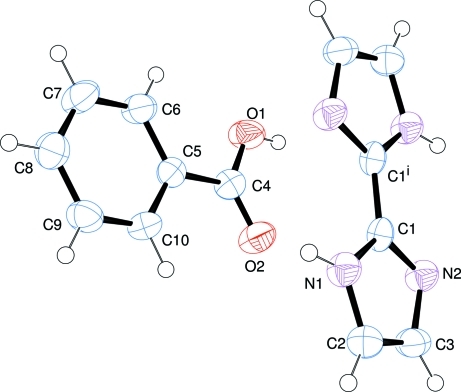
A view of the structure of compound (I) with displacement ellipsoids drawn at the 50% probability level, the biimidazole sits on a center of symmetry passing through the C1—C1 bond. Symmetry code: (i) 1 - x, 1 - y, 1 - z.

**Fig. 2.**
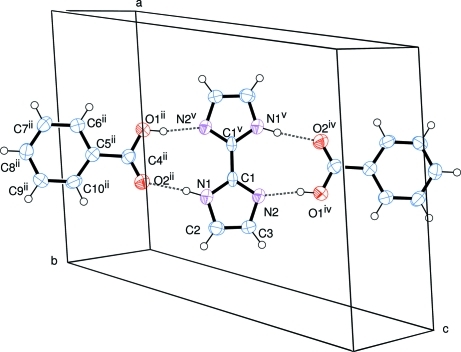
H-bonds (dotting line) in (I). Symmetry codes: (ii) x, 1 + y, z; (iv) 1 - x, -y, 1 - z; (v) 1 - x, 1 - y, 1 - z.

**Fig. 3.**
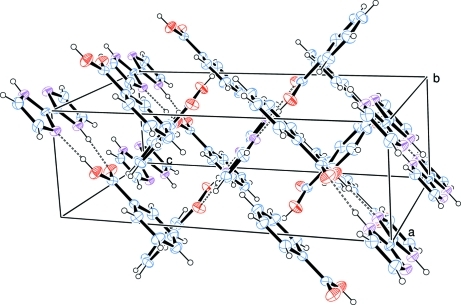
The packing view in the title compound (I), dotting line for H-bonds.

## supplementary crystallographic information

### Comment

Compounds containing the 2,2'-biimidazole moiety have been the focus of several investigations not only due to their biological activity, but also due to their contribution to the field of crystal engineering (Matthews, *et al.* 1990; Tadokoro & Nakasuji, 2000). In these compunds weak interactions, such as C—H···O and C=O···π, play crucial roles in building the overall three-dimensional structure (Mori & Miyoshi, 2004; Li & Yang, 2006; Gao *et al.*,  2009).

The asymmetric unit of compound (I) contains one benzoic acid and 1/2 neutral biimidazole molecule, in which the imidazole rings are coplanar (Fig. 1). Each biimidazole molecule is linked to two benzoic acids *via* strong N—H···O and O—H···N hydrogen bonds (Table 1) twithin planar sheets (Figure 2). These sheets further assemble to layers *via* weak C=O···π (see Table 1, *Cg*1 for centre of N1/C1/N2/C3/C2) interactions between neighboring molecules and arrange alternatively and across along b and *c* axis in two-dimensional structure, and the dihedral angle of the planes are 92.7°. In contrast, two groups of these parallel layers on a twofold rotation axis and inversion centre forming a zigzag conformation along *c* axis in whole three-dimensional network as shown in Fig. 3.

### Experimental

Benzoic acid (0.25 g, 2 mmol) and biimidazole (1 mmol) were dissolved in water(10 ml) by adding 1.4 ml of 2 *M* HCl while stirring. The solutions were stirred for 1 h, then filtered. Filtrate was left to stand at room temperature. Crystals suitable for data collection appeared after a few weeks by slow evaporation of the aqueous solvent.

### Refinement

H atoms attached to C atoms were placed in geometrically idealized positions, with C*sp*^2^ = 0.93 Å, and constrained to ride on their carrier atoms, with *U*_iso_(H) = 1.2*_U_*_eq_(C). H atoms attached to N1 and O1 atoms were located in difference Fourier maps and refined with *U*_iso_(H for N) = 0.06 Å^2^ and U_iso_(H) = 1.5_Ueq_(O); N—H distance is 0.88 (5) Å and the O—H distance is 0.856 Å.

### Crystal data

**Table:**

C_6_H_6_N_4_·2C_7_H_6_O_2_	*F*(000) = 396
*M**_r_* = 378.38	*D*_x_ = 1.367 Mg m^−^^3^
Monoclinic, *P*2_1_/*n*	Mo *K*α radiation, λ = 0.71073 Å
Hall symbol: -P 2yn	Cell parameters from 698 reflections
*a* = 11.232 (5) Å	θ = 2.5–20.8°
*b* = 5.082 (2) Å	µ = 0.10 mm^−^^1^
*c* = 16.342 (7) Å	*T* = 298 K
β = 99.832 (6)°	Block, colorless
*V* = 919.2 (7) Å^3^	0.40 × 0.20 × 0.10 mm
*Z* = 2	

### Data collection

**Table:**

Bruker SMART 1K CCD area-detector diffractometer	1550 independent reflections
Radiation source: fine-focus sealed tube	1243 reflections with *I* > 2σ(*I*)
graphite	*R*_int_ = 0.047
ω scans	θ_max_ = 25.0°, θ_min_ = 2.1°
Absorption correction: multi-scan (*SADABS*; Sheldrick, 2000)	*h* = −13→12
*T*_min_ = 0.962, *T*_max_ = 0.990	*k* = −6→2
3367 measured reflections	*l* = −19→19

### Refinement

**Table:**

Refinement on *F*^2^	Primary atom site location: structure-invariant direct methods
Least-squares matrix: full	Secondary atom site location: difference Fourier map
*R*[*F*^2^ > 2σ(*F*^2^)] = 0.098	Hydrogen site location: inferred from neighbouring sites
*wR*(*F*^2^) = 0.188	H atoms treated by a mixture of independent and constrained refinement
*S* = 1.25	*w* = 1/[σ^2^(*F*_o_^2^) + (0.0425*P*)^2^ + 1.0781*P*] where *P* = (*F*_o_^2^ + 2*F*_c_^2^)/3
1550 reflections	(Δ/σ)_max_ < 0.001
131 parameters	Δρ_max_ = 0.20 e Å^−^^3^
0 restraints	Δρ_min_ = −0.19 e Å^−^^3^

### Special details

**Table:**

Geometry. All e.s.d.'s (except the e.s.d. in the dihedral angle between two l.s. planes) are estimated using the full covariance matrix. The cell e.s.d.'s are taken into account individually in the estimation of e.s.d.'s in distances, angles and torsion angles; correlations between e.s.d.'s in cell parameters are only used when they are defined by crystal symmetry. An approximate (isotropic) treatment of cell e.s.d.'s is used for estimating e.s.d.'s involving l.s. planes.
Refinement. Refinement of *F*^2^ against ALL reflections. The weighted *R*-factor *wR* and goodness of fit *S* are based on *F*^2^, conventional *R*-factors *R* are based on *F*, with *F* set to zero for negative *F*^2^. The threshold expression of *F*^2^ > σ(*F*^2^) is used only for calculating *R*-factors(gt) *etc*. and is not relevant to the choice of reflections for refinement. *R*-factors based on *F*^2^ are statistically about twice as large as those based on *F*, and *R*- factors based on ALL data will be even larger.

### Fractional atomic coordinates and isotropic or equivalent isotropic displacement parameters (Å^2^)

**Table:**

	*x*	*y*	*z*	*U*_iso_*/*U*_eq_	
N1	0.3655 (3)	0.6966 (8)	0.4607 (2)	0.0406 (10)	
H1	0.383 (4)	0.809 (10)	0.423 (3)	0.064 (17)*	
N2	0.3784 (3)	0.3740 (7)	0.5510 (2)	0.0389 (9)	
C1	0.4360 (4)	0.5176 (8)	0.5031 (2)	0.0307 (10)	
C2	0.2533 (4)	0.6694 (10)	0.4821 (3)	0.0436 (12)	
H2	0.1846	0.7679	0.4624	0.052*	
C3	0.2630 (4)	0.4717 (10)	0.5372 (3)	0.0453 (12)	
H3	0.2003	0.4097	0.5625	0.054*	
C4	0.4969 (4)	0.1219 (9)	0.3172 (3)	0.0376 (11)	
C5	0.4964 (4)	0.3271 (9)	0.2521 (2)	0.0347 (10)	
C6	0.5982 (4)	0.3745 (10)	0.2158 (3)	0.0451 (12)	
H6	0.6687	0.2790	0.2329	0.054*	
C7	0.5946 (4)	0.5616 (10)	0.1549 (3)	0.0516 (13)	
H7	0.6625	0.5916	0.1308	0.062*	
C8	0.4913 (4)	0.7050 (10)	0.1294 (3)	0.0491 (13)	
H8	0.4897	0.8321	0.0883	0.059*	
C9	0.3913 (4)	0.6618 (10)	0.1641 (3)	0.0458 (12)	
H9	0.3216	0.7595	0.1468	0.055*	
C10	0.3934 (4)	0.4739 (10)	0.2247 (3)	0.0441 (12)	
H10	0.3244	0.4447	0.2478	0.053*	
O1	0.5977 (3)	−0.0029 (7)	0.3367 (2)	0.0532 (10)	
H1A	0.5971	−0.1186	0.3747	0.080*	
O2	0.4086 (3)	0.0801 (7)	0.3491 (2)	0.0517 (9)	

### Atomic displacement parameters (Å^2^)

**Table:**

	*U*^11^	*U*^22^	*U*^33^	*U*^12^	*U*^13^	*U*^23^
N1	0.043 (2)	0.037 (2)	0.042 (2)	−0.0004 (19)	0.0097 (18)	0.009 (2)
N2	0.041 (2)	0.035 (2)	0.043 (2)	−0.0011 (18)	0.0116 (17)	0.0088 (19)
C1	0.044 (2)	0.022 (2)	0.027 (2)	0.003 (2)	0.0077 (19)	0.0055 (19)
C2	0.037 (3)	0.048 (3)	0.046 (3)	0.004 (2)	0.007 (2)	0.000 (3)
C3	0.032 (2)	0.057 (3)	0.048 (3)	0.000 (2)	0.009 (2)	0.011 (3)
C4	0.041 (3)	0.029 (2)	0.043 (3)	−0.004 (2)	0.009 (2)	−0.002 (2)
C5	0.038 (2)	0.032 (3)	0.034 (2)	−0.004 (2)	0.0064 (19)	−0.005 (2)
C6	0.037 (3)	0.046 (3)	0.053 (3)	0.002 (2)	0.012 (2)	0.008 (3)
C7	0.048 (3)	0.052 (3)	0.060 (3)	−0.001 (3)	0.024 (2)	0.013 (3)
C8	0.051 (3)	0.048 (3)	0.049 (3)	−0.003 (3)	0.008 (2)	0.013 (3)
C9	0.036 (3)	0.046 (3)	0.054 (3)	0.006 (2)	0.004 (2)	0.009 (3)
C10	0.033 (2)	0.055 (3)	0.046 (3)	−0.005 (2)	0.013 (2)	0.002 (3)
O1	0.0371 (17)	0.058 (2)	0.065 (2)	0.0067 (18)	0.0089 (15)	0.0244 (19)
O2	0.0465 (19)	0.051 (2)	0.063 (2)	0.0094 (17)	0.0220 (16)	0.0155 (18)

### Geometric parameters (Å, °)

**Table:**

N1—C1	1.322 (5)	C5—C10	1.385 (6)
N1—C2	1.371 (5)	C5—C6	1.397 (6)
N1—H1	0.88 (5)	C6—C7	1.371 (6)
N2—C1	1.319 (5)	C6—H6	0.9300
N2—C3	1.370 (5)	C7—C8	1.374 (6)
C1—C1^i^	1.469 (8)	C7—H7	0.9300
C2—C3	1.342 (6)	C8—C9	1.359 (6)
C2—H2	0.9300	C8—H8	0.9300
C3—H3	0.9300	C9—C10	1.373 (6)
C4—O2	1.216 (5)	C9—H9	0.9300
C4—O1	1.289 (5)	C10—H10	0.9300
C4—C5	1.489 (6)	O1—H1A	0.8564
			
C1—N1—C2	106.9 (4)	C6—C5—C4	121.4 (4)
C1—N1—H1	129 (3)	C7—C6—C5	120.1 (4)
C2—N1—H1	124 (3)	C7—C6—H6	119.9
C1—N2—C3	104.4 (4)	C5—C6—H6	119.9
N2—C1—N1	112.4 (4)	C6—C7—C8	120.5 (4)
N2—C1—C1^i^	124.0 (5)	C6—C7—H7	119.8
N1—C1—C1^i^	123.6 (5)	C8—C7—H7	119.8
C3—C2—N1	105.9 (4)	C9—C8—C7	120.2 (5)
C3—C2—H2	127.0	C9—C8—H8	119.9
N1—C2—H2	127.0	C7—C8—H8	119.9
C2—C3—N2	110.4 (4)	C8—C9—C10	120.0 (4)
C2—C3—H3	124.8	C8—C9—H9	120.0
N2—C3—H3	124.8	C10—C9—H9	120.0
O2—C4—O1	123.6 (4)	C9—C10—C5	121.2 (4)
O2—C4—C5	121.7 (4)	C9—C10—H10	119.4
O1—C4—C5	114.7 (4)	C5—C10—H10	119.4
C10—C5—C6	118.0 (4)	C4—O1—H1A	113.7
C10—C5—C4	120.6 (4)		
			
C3—N2—C1—N1	0.0 (5)	O1—C4—C5—C6	0.5 (6)
C3—N2—C1—C1^i^	−179.6 (5)	C10—C5—C6—C7	0.0 (7)
C2—N1—C1—N2	0.0 (5)	C4—C5—C6—C7	178.9 (4)
C2—N1—C1—C1^i^	179.6 (5)	C5—C6—C7—C8	0.4 (7)
C1—N1—C2—C3	0.0 (5)	C6—C7—C8—C9	−0.3 (8)
N1—C2—C3—N2	0.0 (5)	C7—C8—C9—C10	−0.1 (7)
C1—N2—C3—C2	0.0 (5)	C8—C9—C10—C5	0.5 (7)
O2—C4—C5—C10	−1.3 (6)	C6—C5—C10—C9	−0.4 (7)
O1—C4—C5—C10	179.3 (4)	C4—C5—C10—C9	−179.3 (4)
O2—C4—C5—C6	179.8 (4)		

Symmetry codes: (i) −*x*+1, −*y*+1, −*z*+1.

### Hydrogen-bond geometry (Å, °)

**Table:**

Cg1 is the centroid of the [please define] ring.

**Table:**

*D*—H···*A*	*D*—H	H···*A*	*D*···*A*	*D*—H···*A*
O1—H1A···N2^ii^	0.86	1.77	2.613 (5)	170
N1—H1···O2^iii^	0.88 (5)	1.89 (5)	2.767 (5)	173 (5)
C4—O2···Cg1	1.216 (5)	3.674 (2)	4.388 (2)	118.37 (6)

Symmetry codes: (ii) −*x*+1, −*y*, −*z*+1; (iii) *x*, *y*+1, *z*.
